# The integrin CD11b inhibits MSU-induced NLRP3 inflammasome activation in macrophages and protects mice against MSU-induced joint inflammation

**DOI:** 10.1186/s13075-024-03350-5

**Published:** 2024-06-11

**Authors:** Driss Ehirchiou, Ilaria Bernabei, Vishnuprabu Durairaj Pandian, Sonia Nasi, Veronique Chobaz, Mariela Castelblanco, Alexander So, Fabio Martinon, Xiaoyun Li, Hans Acha-Orbea, Thomas Hugle, Li Zhang, Nathalie Busso

**Affiliations:** 1 Service of Rheumatology, Department of Musculoskeletal Medicine, Centre Hospitalier Universitaire Vaudois, University of Lausanne, Lausanne, Switzerland; 2 Department of Biochemistry, University of Lausanne, Epalinges, Switzerland; 3 Department of Fundamental Oncology, University of Lausanne, Lausanne, Switzerland; 4 Department of Physiology, Center for Vascular and Inflammatory Diseases, University of Maryland School of Medicine, Baltimore, MD United States

**Keywords:** CD11b, Integrin, NLRP3 inflammasome, MSU crystals, Animal model, CD11b agonist

## Abstract

**Objective:**

In gout, monosodium urate crystals are taken up by macrophages, triggering the activation of the NLRP3 inflammasome and the maturation of IL-1β. This study aimed to investigate the role of integrin CD11b in inflammasome activation in macrophages stimulated by MSU.

**Methods:**

BMDM from WT and CD11b KO mice were stimulated in vitro with MSU crystals. Cellular supernatants were collected to assess the expression of the inflammatory cytokines by enzyme-linked immunosorbent assay and western blot methods. The role of integrin CD11b in MSU-induced gouty arthritis in vivo was investigated by intra-articular injection of MSU crystals. Real-time extracellular acidification rate and oxygen consumption rate of BMDMs were measured by Seahorse Extracellular Flux Analyzer.

**Results:**

We demonstrate that CD11b-deficient mice developed exacerbated gouty arthritis with increased recruitment of leukocytes in the joint and higher IL-1β levels in the sera. In macrophages, genetic deletion of CD11b induced a shift of macrophage metabolism from oxidative phosphorylation to glycolysis, thus decreasing the overall generation of intracellular ATP. Upon MSU stimulation, CD11b-deficient macrophages showed an exacerbated secretion of IL-1β. Treating wild-type macrophages with a CD11b agonist, LA1, inhibited MSU-induced release of IL-1β *in vitro* and attenuated the severity of experimental gouty arthritis. Importantly, LA1, was also effective in human cells as it inhibited MSU-induced release of IL-1β by peripheral blood mononuclear cells from healthy donors.

**Conclusion:**

Our data identified the CD11b integrin as a principal cell membrane receptor that modulates NLRP3 inflammasome activation by MSU crystal in macrophages, which could be a potential therapeutic target to treat gouty arthritis in human patients.

**Supplementary Information:**

The online version contains supplementary material available at 10.1186/s13075-024-03350-5.

## Introduction

Gout is an inflammatory joint disorder that results from the deposition of monosodium urate crystals (MSU) in joints of patients with hyperuricemia. According to the current understanding of the pathogenesis of acute gout, MSU activate resident articular cells (for example macrophages) during the initiation phase. The activation of resident cells by MSU induces the synthesis of several inflammatory mediators [1–3]. Both in humans and in murine models, IL-1β, IL-6, and IL-8 are the main mediators implicated in the onset of acute gout and IL-1β inhibition or deficiency during onset of gout results in effective suppression of joint swelling and influx of inflammatory cells [4, 5].

It is well known that the activation of the nucleotide-binding oligomerization domain-like receptor protein 3 (NLRP3) inflammasome is critical in both the initiation phase and the progression of gout [6]. NLRP3 inflammasome is a multiprotein cytoplasmic complex formed by the NLRP3 sensor molecule, the adaptor protein ASC, and the effector caspase-1 [7] [8]. NLRP3 inflammasome activation needs two independent steps *i.e.*, priming and activation. Priming step, which can be induced by toll-like receptors (TLRs), is required to increase the transcription of basal expression of NLRP3 protein and the pro-form of IL-1 β (pro-IL-1β) [9]. The activation step occurs once the primed cells recognize MSU crystals within the joint. Upon MSU crystal exposure, activation of the NLRP3 inflammasome leads to protease caspase-1 generation and ultimately processing of pro-IL-1β into bioactive IL-1β [1]. Although there is a growing body of evidence supporting the mechanism and efficacy of NLRP3 inflammasome inhibitors in experimental gout [10], no approved human agents are available.

Many mechanisms that mediate NLRP3 inflammasome activation by MSU crystals have been identified including reactive oxygen species (ROS), ATP and potassium ion efflux. Accumulated evidence suggested a link between inflammasome and the metabolic signature of macrophages [11, 12]. Indeed, it has been reported that, when macrophages are triggered to adopt an inflammatory profile, their metabolic profile changes, switching from oxidative to glycolytic metabolism [13, 14]. While the stimuli and the mechanisms involved in NLRP3 inflammasome activation are well documented, the molecules and the molecular mechanisms involved in the negative control of NLRP3 inflammasome activation remain to be further elucidated.

CD11b integrin (CD11b/CD18, Mac‐1, or α_M_β_2_) is a member of the beta 2 integrin family of adhesion receptors expressed on most immune cells, including monocytes/macrophages, neutrophils and dendritic cells [15]. A wide range of ligands have been described for CD11b integrin, including complement activation fragments (C3b/iC3b), intravascular adhesion molecule-1 (ICAM-1, CD154), fibrinogen, and high mobility group box protein 1 (HMGB-1) [4]. CD11b integrin has been shown to contribute to cell activation, chemotaxis, cytotoxicity, phagocytosis and tolerance induction [15]. More recently, there is considerable scientific rationale to support the hypothesis that CD11b activation is of therapeutic potential in a range of inflammatory disease situations.

Pro - and anti-inflammatory effects of CD11b in monocytes and macrophages have been reported [16], which can impact the appropriate immune responses. Therefore, dysregulated integrin expression, ligand binding, receptor activation or signaling can favor autoimmune and inflammatory diseases. On the one hand, CD11b activation can promote the proinflammatory response of macrophages, most likely via its binding of fibrinogen. Genetic deletion of CD11b or mutation of the CD11b-recognition site in fibrinogen has been reported to reduce the development of several inflammatory diseases, including stroke, multiple sclerosis, and Alzheimer’s Disease [17, 18]. On the other hand, CD11b also possesses anti-inflammatory properties. It inhibits the proinflammatory function of dendritic cells [19, 20] and enhances their tolerogenic activities [21]. Moreover, CD11b suppresses TLR-dependent inflammation [22] and Th17 differentiation [23], as well as negatively regulates BCR signaling via the Lyn-CD22-SHP-1 inhibitory pathway [24]. Most importantly, independent GWAS studies have identified a number of loss-of-function variants (R77H, P1146S and A858V) of CD11b that are strongly linked to an increased risk of systemic lupus erythematosus in human patients [25–27] further underscoring the critical role of CD11b in immunosuppression. Indeed, genetic deletion of CD11b in mice has been shown to abolish the development of systemic immune tolerance [23], worsens glomerulonephritis [28], exacerbates rheumatoid arthritis [29], and intensifies soluble immune complex (IC)-mediated organ damage [30].

In line with the anti-inflammatory effect of CD11b, a small allosteric agonist of CD11b, Leukadherin‐1 (LA1), decreased inflammation in animal models of pancreatic cancer, nephritis and chronic lung disease [31–34].

In this study, we sought to evaluate the role of CD11b in NLRP3 inflammasome activation and the underlying mechanisms, and to test whether acute joint inflammation can be treated with the CD11b agonist. We show that lack of CD11b-mediated metabolic changes in CD11b-deficient macrophages is associated with excessive IL-1 and IL-6 production *in vitro*, and with exacerbated gouty arthritis *in vivo*. Furthermore, we describe that a CD11b agonist represents a highly potent NLRP3 inflammasome inhibitor that can efficiently suppress joint inflammation in a mouse model of gouty arthritis.

## Materials and methods

### Mice and induction of MSU crystal-induced arthritis

CD11b KO mice (C57BL/6J background) were provided by Prof. Britta Engelhardt (Theodor Kocher Institute, University of Bern). For experiments, 10-12 weeks old female CD11b-/- and as controls, age-matched female CD11b+/+ were used. All animals were kept in a temperature-controlled environment in a ventilated rack with a 12:12-h light: dark cycle. Food and water were given *ad libitum*. Gouty arthritis was induced in female mice by injecting into mice knees 10 μl of a mixture of 300 μg of MSU crystals, 200 μM C16:0 palmitic acid (Sigma-Aldrich), and 1 mg of bovine serum albumin (Sigma-Aldrich) as described before [35]. Vehicle-injected knees served as controls. The effect of LA1 (ChemBridge, dissolved in DMSO) tested at 3.9 mg/kg in the gouty arthritis model by intravenous injection performed 30 min before intra-articular injection of MSU crystals. Mice injected *i.v.* with vehicle only were used as controls. The dose of LA1 was selected according to a previous study [36]. For all experiments, animals were sacrificed 6 h after crystal exposure.

### Histological analysis

The entire knee joint was removed and fixed in 10% formaldehyde for 7 days before decalcification using 5% formic acid and processed for paraffin embedding. Tissue sections (7 μm) were stained with H&E. Histopathological changes in the knee joints were scored in the patellar/femoral region in five semi-serial sections by the number of infiltrating cells in the synovial lining and/or joint cavity on a scale ranging from 0 to 3 where 0 = no inflammation, 1 = mild inflammation, 2 = moderate inflammation, and 3 = severe inflammation. Joint inflammation was graded on decoded slides by two separate observers.

### THP-1 cell culture

THP-1 cells were procured from ATCC (TIB-202) and cultured in RPMI-1640 medium supplemented with 10% fetal bovine serum (FBS) and 0.05mM 2-mercaptoethanol. Lenti-X-293T Cell Line (Takara) was used for production of lentiviral particles, and this cell line was cultured in DMEM supplemented with 10% FBS. Both THP-1 and Lenti-X-293T cells were grown in 5% CO2 at 37°C throughout the procedures and experiments.

### Lentiviral CD11b-CRISPR knockout

#### Generation of THP-1-Cas9 cells

LentiX-293T cells were grown in T75 flask to 80% confluency, transfection cocktail was made in 1 ml of Optimem (Gibco) with 40 μl of Xtremegene-HP (Roche) mixed with the plasmids; Lenticas9-Blast (Addgene# 52962) (6µg), psPAX2 (Addgene# 12260) (5 µg) and pMD2.G (Addgene# 12259) (2.5 µg). Transfection mix incubated in room temperature for 20min and added to the cells. Post-transfection, 48h, 72h and 96h culture media was collected and pooled. Lentiviral particle were concentrated using 40% sucrose centrifugation at 10,000xg for 4h at 4°C. The lentiviral particle pellet was resuspended in 250 μl sterile PBS and used to infect the THP-1 cells (0.3 million cells) with 8μg/ml Polybrene. Twenty-four hours post infection, fresh media was added to the cells, which were allowed to grow for 2-3 days, and Blasticidin selection (10 μg/ml) was done for 3-5 days. Post blasticidin selection, cells were grown in normal media and screened for the Cas-9 expression using q-RT-PCR and Western Blot. These THP-1 Cas9 (THP-1-WT) cells were used for further experiments.

#### Generation of THP-1 CD11b KO cells

A 20bp guide RNA sequence (tttgcgatcg agg gtgagtc agg(PAM)) (chr16:31275687-31275709) was designed at the end of exon9 (of CD11b mRNA) targeting the splice site. The guide RNA was cloned in the lentiGuide-Puro plasmid (addgene – 52963) as per the protocol by Sanjana N.E et al [37, 38] and Lentiviral particles with guide RNA were produced as described earlier in LentiX-293T, where the lentiGuide-Puro-CD11bsgRNA plasmid was used with psPAX2 and pMD2.G. THP-1 Cas9 cells were infected with the concentrated lentiviral particles and puromycin selection (5µg/ml) was performed for 5 days. Post puromycin selection, cells were grown in normal media and screened for CD11b expression by flow-cytometry. The CD11b negative cells (THP-1 CD11bKO) were sorted and collected for further experiments.

#### CD11b rescue of THP-1 CD11bKO cells

The coding DNA sequence (CDS) of ITGAM (NM_001145808) was cloned in pLVX-M-puro vector (Addgene# 125839). Lentiviral particles with CD11b CDS were produced as described earlier in LentiX-293Tcells, where the pLVX-M-Puro-CD11b plasmid was used with psPAX2 and pMD2.G. THP-1 CD11bKO cells were infected with the concentrated lentiviral particles and puromycin selection (5 µg/ml) was performed for 5 days. Post puromycin selection, cells were grown in normal media and screened for CD11b expression by flow-cytometry and western blotting. These CD11b-rescued cells (THP-1-CD11b rescued) were used for further experiments

### THP-1 cell stimulation

On day-1, THP-1-WT, THP-1-CD11b KO and THP-1-CD11b rescued cells were differentiated with 10nM phorbol 12-myristate 13-acetate (PMA) (Sigma). On day-2, PMA was removed and cells were rested in normal complete media for 24h. On day-3, cells were treated with Pam3CSK4 (Invivogen) at a concentration of 100 ng/ml in complete media for overnight. On day-4, cells were washed with serum free media and were treated with 500 µg/ml mono-sodium urate (MSU) crystals, in presence or absence of the NLRP3 inflammasome inhibitor MCC50 (10 μM), in serum free media for 6h. Six hours following MSU treatment, the culture media was collected and frozen at -80°C and later used for ELISA.

### Preparation of BMDMs

Bone marrow cells were isolated from the tibia and femurs of 10–12-week–old WT and CD11b KO mice. For differentiation into Bone marrow derived macrophages (BMDMs), the isolated cells were incubated for 7 days on Petri dishes in Dulbecco's modified Eagle's medium (DMEM, Life Technologies) with 30% L929 conditioned medium (source of M-CSF), 10% FBS (PAA Laboratories GmbH, Austria), 1% HEPES (Life Technologies), and 1% penicillin/streptomycin (Life Technologies). After differentiation, cells were detached using cold PBS, and plated for stimulation experiments.

### BMDM and PBMC stimulation

Murine BMDMs or human PBMCs were plated in 96-well dishes at a density of 1 × 10^5^ cells/well. Cells were primed overnight with 100 ng/ml of Pam3CSK4 (Invitrogen) using DMEM with 10% FBS, 1% HEPES, and 1% penicillin/streptomycin. Then primed cells were stimulated in serum-free medium with MSU crystals (500 μg/ml) for 6h. LA1, at different concentrations in 0.1% DMSO or vehicle alone as control, were incubated 30 min before MSU crystal stimulation.

### ELISA

Mouse and human IL-1β and IL-6 ELISA kits (eBioscience, Inc., San Diego, CA) were used to measure the corresponding cytokine levels in the supernatants according to the manufacturer's instructions.

### Real Time Quantitative PCR Analysis

Cells were treated with TRIzol (500 μl for 1 million cells) and RNA was extracted (RNA Clean and Concentrator 5-Zymoresearch) and reverse transcribed (Superscript II- Invitrogen^TM^). Quantitative Real Time PCR (qRT-PCR) was performed with gene specific using the LightCycler^®^ 480 system (Roche Applied Science). Normalization was performed against Gapdh reference gene, and increase of transcripts was calculated against control cells. The following primers were used: glucose 6-phosphate (G6PD2), F 5'-GTG GAG AAA CCC TCC GGG AG-3′, R 5'-TCA AAA TAG CCC CCA CGA CC-3′; aldolase A (Aldoa), F 5'-AGG CCA TGC TTG CAC TCA G-3′, R 5'-AGG GCC CAG GGC TTC AG-3′; lactate dehydrogenase A (LDHA), F 5'-GGT TGG TGC TGT TGG CAT GC-3′, R 5'-TGC CCC AGC CGT GAT AAT GA-3′. mGapdh, F 5'- CTC ATG ACC ACA GTC CAT GC-3′, R 5'- CAC ATT GGG GGT AGG AAC AC-3′.

### Seahorse extracellular flux analysis

Real-time extracellular acidification rate (ECAR) and oxygen consumption rate (OCR) of BMDMs were measured by an XF96 Seahorse Extracellular Flux Analyzer following the manufacturer's instruction. BMDMs (1x10^5^) were plated in a Seahorse Bioscience culture plate for overnight. The ECAR and OCR were measured in response to glucose (10 mM), oligomycin (1 μM) and 2-DG (50 mM) for the Glycolysis Stress assay and oligomycin (1 μM), carbonyl cyanide-4-(trifluoromethoxy) phenylhydrazone (FCCP, 1 μM) and rotenone/antimycin A (Rot/AA, 0.5 μM) for the Cell Mito Stress assay. Three measurements were made under basal conditions. Each condition was performed with 4–6 replicates, and the readings of OCR and ECAR of each well were normalized to protein amount.

### Metabolomics study

To extract intracellular metabolites from BMDMs, the previously published protocol was employed [39]. Cells were washed with 2 mL of ice-cold ammonium carbonate buffer (75mM) twice on ice and were lysed quickly in 500ul of 70% ethanol solution. The metabolite extractions were prepared by spinning the samples at 14,000 rpm for 10 min at 4 °C and were stored at −80 °C Samples were subsequently analyzed using direct flow-injection mass-spectrometry method to profile relative changes of metabolite concentrations (General Metabolics, LCC, Boston, USA). Specifically, samples were injected into an Agilent 6550 Series Quadrupole Time-of-flight mass spectrometer (Agilent, Santa Clara, CA), operated in negative mode. The experiment was performed in triplicates and each sample was injected twice. From the spectrum of detected ions, amino acids, purine, pyrimidine and lipid metabolites were annotated. All steps of mass spectrometry data processing and analysis were performed with MATLAB (The Mathworks, Natick, MA, USA) using. The resulting data included the intensity of each mass peak in each analyzed sample. Peak picking was done for each sample once on the total profile spectrum obtained by summing all single scans recorded over time and using wavelet decomposition as provided by the Bioinformatics toolbox. In this procedure, a cutoff was applied to filter peaks of less than 5,000 ion counts (in the summed spectrum) to avoid detection of features that are too low to deliver meaningful insights. Centroid lists from samples were then merged to a single matrix by binning the accurate centroid masses within the tolerance given by the instrument resolution. The resulting matrix lists the intensity of each mass peak in each analyzed sample. An accurate common m/z was recalculated with a weighted average of the values obtained from independent centroiding. Averages of triplicates were calculated for comparisons between groups, and statistical analysis with pFDR (false discovery rate) values was performed. Manual annotation of metabolites pathways was done by using HMDB [40] and KEGG [41]. Heatmaps were created by use of heatmap.2 function in Rstudio (RStudio Team (2020). RStudio: Integrated Development for R. RStudio, PBC, Boston, MA URL http://www.rstudio.com), by inputting fold changes between comparisons and calculated pFDR values (* p<0.05).

### Assay of phagocytosis

WT and CD11b KO BMDM were treated with MSU crystals for 4 h at 37°C and then washed 3 times with PBS containing 5 mM EDTA and harvested in the same buffer. The proportion of macrophages taking up MSU crystals was assessed by flow cytometry based on increased side scatter properties. All data acquisition was performed on LSRII cytometer using FACS Diva6 (Becton Dickinson) and FlowJoX software for data processing.

### LDH Measurement

Measurement of lactate dehydrogenase (LDH) as a means of cytotoxicity assay was performed with using CytoTox-ONE Homogeneous Membrane Integrity Assay (Promega) according to the manufacturer’s instructions. Values were calculated as percentage of cytotoxicity using the following formula: LDH release (%) = [(value in sample) — (background)]/[(value in Triton X-100-treated sample) — (background)] x100.

### Reactive Oxygen Species Level Measurement

Mitochondrial ROS level was measured with Red Mitochondrial Superoxide Indicator (MitoSOX, Life Technologies). Briefly, primed BMDMs were stimulated for 2 h with 500 μg/ml MSU crystals and then loaded 30 min with MitoSOX (at 5 μM final concentration), and fluorescence intensity measured (excitation 510 nm, emission 580 nm) using the Spectrax M5e reader.

### Measurement of intracellular ATP

Cells were lysed in a buffer containing 20 mm HEPES, pH 7.5, 0.5% Nonidet P-40, 50 mm KCl, 150 mm NaCl, 1.5 mm MgCl_2_, 1 mm EGTA . Intracellular ATP (iATP) was measured using CellTiter-Glo luminescent cell viability assay (Promega, Madison, WI) according to the manufacturer’s instructions. Luminescence was measured with a luminometer and ATP concentration was calculated by using an ATP standard curve. Protein concentration was determined by the BCA method and iATP content was expressed as nanomoles per mg protein.

### Immunoblot

Cells were lysed in a buffer containing 20 mm HEPES, pH 7.5, 0.5% Nonidet P-40, 50 mm KCl, 150 mm NaCl, 1.5 mm MgCl_2_, 1 mm EGTA and protease inhibitors. Cell culture supernatants were precipitated by methanol/chloroform. Samples were then immunoblotted using the following antibodies from Adipogen: anti-NLRP3 (AG-20B-0014), anti- caspase-1 (AG-20B-0042-C100), anti ASC (AG-25B-006) and anti-tubulin (AG-27B-0005-C100). Goat anti-mouse IL-1β antibody (AF-401-NA) was from R&D Systems.

### Statistical Analysis

All values are expressed as the mean ± SD. Variation between data sets was evaluated using the Student’s *t*-test or one-way or two-way ANOVA test, where appropriate. Differences were considered statistically significant for a value of *p* < 0.05 (^∗^*p* < 0.05, ^∗∗^*p* < 0.01, ^∗∗∗^*p* < 0.001, ^****^p < 0.0001). Data was analyzed with GraphPad Prism software (GraphPad software).

### Ethics Statement

Experiments on mice were performed in strict accordance with the Swiss Federal Regulations and approved by the “Service de la consommation et des affaires vétérinaires du Canton de Vaud*,”* Switzerland. All efforts were made to minimize suffering and minimize the number of mice needed to assess statistical significance and experimental reproducibility. Human samples were obtained with the approval of the Centre Hospitalier Universitaire Vaudois ethical committee and patients’ written informed consent.

## Results

### CD11b deficiency exacerbated pro-inflammatory cytokine production in macrophages following MSU crystal stimulation

**Fig. 1:**
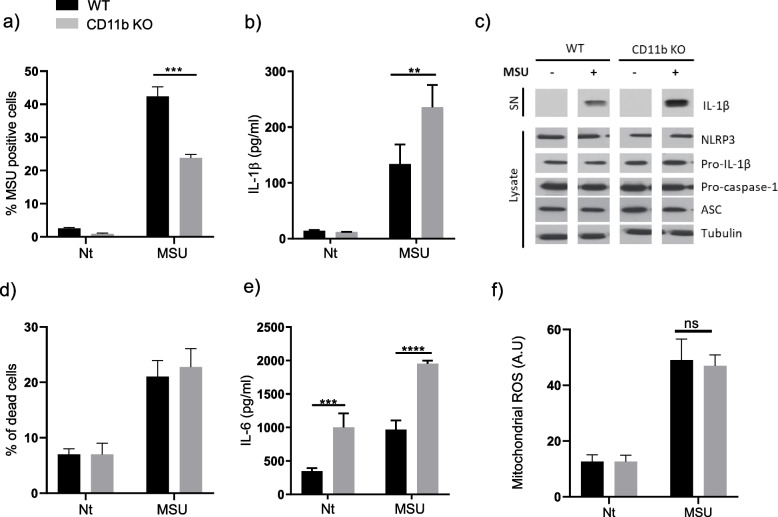
CD11b deficiency exacerbated NLRP3 inflammasome activation in murine macrophages following MSU crystal stimulation. **a** BMDMs were incubated with 500 μg/ml MSU for 6h and the percentage of BMDMs that took up the MSU crystals was analyzed by flow cytometry based on the increase in the side scatter intensity profile. **b** and **c** WT or CD11b KO BMDMs primed overnight with 100 ng/ml of Pam3Cys were stimulated for 6 hours with 500 μg/ml of MSU. IL-1β (**b**) and IL-6 (**e**) concentrations were determined by ELISA in cell supernatants. **c** Western-blot analysis of NLRP3 inflammasome components in supernatants (SN) and in cell lysates of 6 hours MSU-stimulated primed BMDM from WT and CD11b KO mice. **d** LDH release in cell supernatants from point (**b**). **f** Primed BMDMs from WT mice or CD11b-deficient were stimulated with 500 μg/ml of MSU for 2 h. Mitochondrial ROS was measured using MitoSOX. Results in a, b, d, e, and f are expressed as mean ± SD. Data shown represent mean ± SD with group size *n*=3-6

The role of CD11b in NLRP3 inflammasome activation was first examined using macrophages generated *in vitro* from WT and CD11b-deficient mice. As phagocytosis of crystals is a prerequisite for crystal-induced inflammasome activation, we first compared phagocytic efficiency of WT and CD11b-deficient macrophages using flow cytometric analysis. The results show that CD11b-deficient macrophages exhibited lower phagocytic activity toward MSU in comparison to WT macrophages (Fig. 1a). We next evaluated NLRP3 inflammasome activation by MSU crystals in WT and CD11b-deficient cells. In CD11b-deficient murine macrophages MSU significantly increased secretion of IL-1β compared to WT macrophages (Fig. 1b). We performed a Western-blot analysis to assess the levels of inflammasome components (Fig. 1c). In line with the ELISA Fig. 1b we found increased IL-1β (p17 kDa) in MSU-stimulated BMDM supernatants from CD11b KO mice, whereas the levels of ASC, procaspase-1, pro-IL-1β and NLRP3 in cells were similar between WT and KO BMDM. Increased IL-1β secretion was not accounted for by cell death as cell survival was similar in WT and CD11b KO cells (Fig. 1d). We also measured IL-6 secretion, which was exacerbated in CD11b KO cells in both in unstimulated and MSU crystal-stimulated conditions (Fig. 1e), suggesting that the mechanisms involved in exacerbation of IL-1β and IL-6 secretion are different (IL-1β secretion being only enhanced in the stimulated condition). As previous studies have reported that mitochondrial ROS contributes to the activation of NLRP3 inflammasome [42], we speculated that CD11b deficiency could favor ROS production and NLRP3 inflammasome activation. However, we obtained similar mitochondrial ROS production in WT and CD11b deficient cells (Fig. 1f). Together, these results indicate that CD11b deficiency enhanced NLRP3 inflammasome activation by MSU crystals independently of ROS production and despite lower phagocytic activity.

**Fig. 2:**
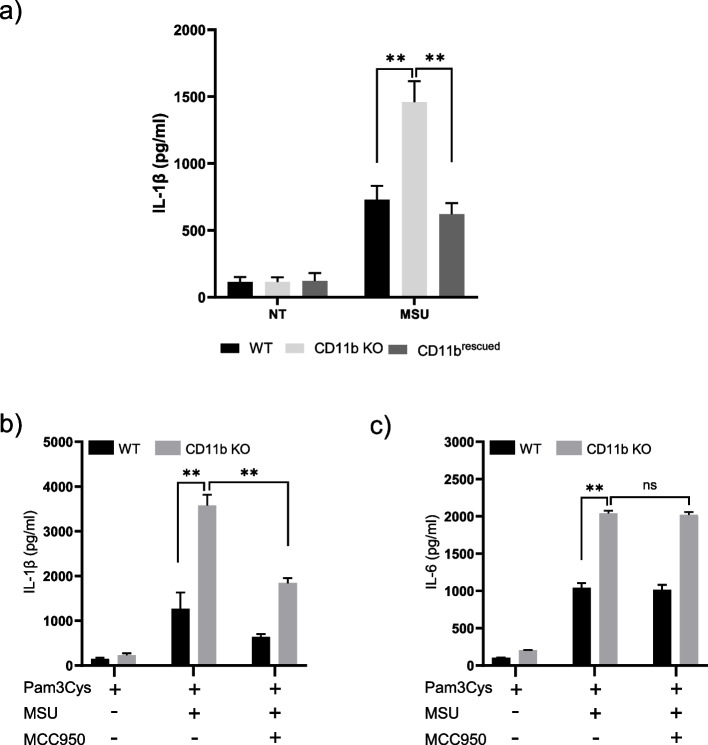
CD11b deficiency exacerbated NLRP3 inflammasome activation in human THP-1 macrophages following MSU crystal stimulation. Differentiated THP1-Cas9 (WT), THP1-CD11b KO (KO) and THP1-CD11b rescued cells (CD11b rescued) were primed with Pam3CSK4 overnight and then stimulated with MSU for 6 hours. The amount of human IL-1β secreted in the culture media was measured by ELISA. Data shown represent mean ± SD with group size *n*=4. **b** and **c** differentiated THP1-WT and THP1- CD11b KO cells were primed with Pam3CSK4 (100ng/ml) for 16h and then treated with MSU (500µg/ml) for 6h, with or without with MCC950 (10µM). Production of IL-1β (b) and IL-6 (c) in cell culture supernatants was measured by ELISA. Data shown represent mean ± SD with group size *n*=4

To confirm the role of CD11b in NLRP3 inflammasome activation by MSU crystals in human macrophages, CD11b was knocked out in human THP-1 cells using the Cas9/CRISPR system. Compared with THP-1 WT, THP-1 CD11bKO cells showed enhanced IL-1β secretion upon MSU stimulation (Fig. 2a). To validate the causative relationship between the increasing secretion of IL-1β and the absence of CD11b on THP-1 cells, we rescued the THP-1 CD11b KO cells by lentiviral CD11b expression. As expected, re-expression of CD11b in THP-1 CD11bKO cells reduced the secretion of IL-1β upon MSU stimulation (Fig. 2a). To gain insights into the mechanisms involved in exacerbation of IL-1β in THP-1-CD11b KO cells, we investigated the impact of the NLRP3 inflammasome inhibitor MCC950 (Fig. 2b). As expected, IL-1β secretion was increased in MSU-stimulated THP-1-CD11 KO cells compared to WT cells. Importantly, in presence of MCC950, there was a significant 2-fold reduction of secreted IL-1β in both cell lines . By contrast, in the same conditions MCC950 had no effect on IL-6 (Fig. 2c). Taken together these results suggest that CD11b deficiency exacerbated pro-inflammatory cytokines release in macrophages following MSU crystal stimulation in NLRP3-inflammasome dependent (IL-1β) and independent way (IL-6).

### CD11b-deficient mice displayed enhanced joint inflammation in a model of MSU-induced gouty arthritis

**Fig. 3:**
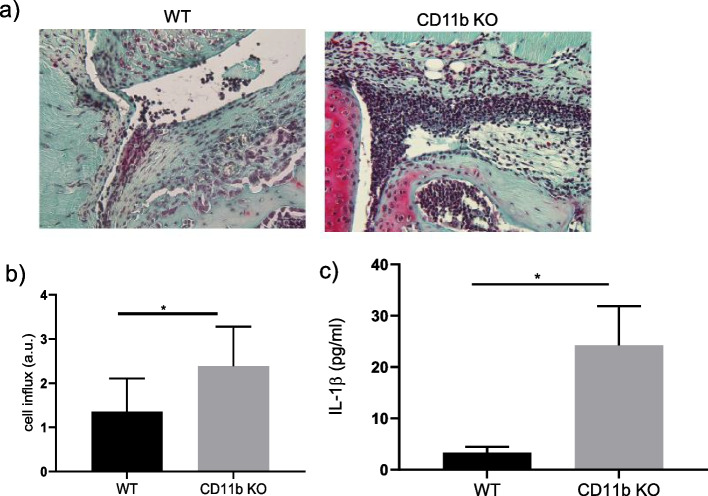
CD11b deficiency exacerbated local and systemic inflammation in MSU-induced gouty arthritis. a) Representative images of H&E-stained histological knee sections from WT and CD11b KO mice injected i.a. with 300 μg of MSU crystals for 6 h. **b** Histological sections in (**a**) were scored for cell infiltration using an arbitrary scale from 0 to 3 and expressed as mean±SD of at least 7 mice per group. **c** IL-1β production in the sera of mice injected with MSU crystals for 6h. Data show mean ± SD of 8-11 mice per group

Given that CD11b inhibits NLRP3 inflammasome activation *in vitro*, we further examined the role of this integrin in MSU-induced gouty arthritis *in vivo* using a well-characterized mouse model [35]. Intra-articular cell influx was assessed 6 h after injection of MSU into the knee joints of WT and CD11b-deficient mice. H&E staining revealed significantly more infiltrating cells in the knee joints of CD11b-deficient mice as compared to WT control mice (Fig. 3a and semi-quantitative scoring Fig. 3b). The pro-inflammatory profile of CD11b-deficient mice was further supported by the significantly higher level of IL-1β in the serum obtained from these mice (Fig. 3c). Taken together, these results suggest that CD11b inhibits NLRP3 inflammasome activation and IL-1β secretion *in vivo* in the context of gouty inflammation.

### CD11b deficiency impacts metabolic pathways in macrophages

**Fig. 4:**
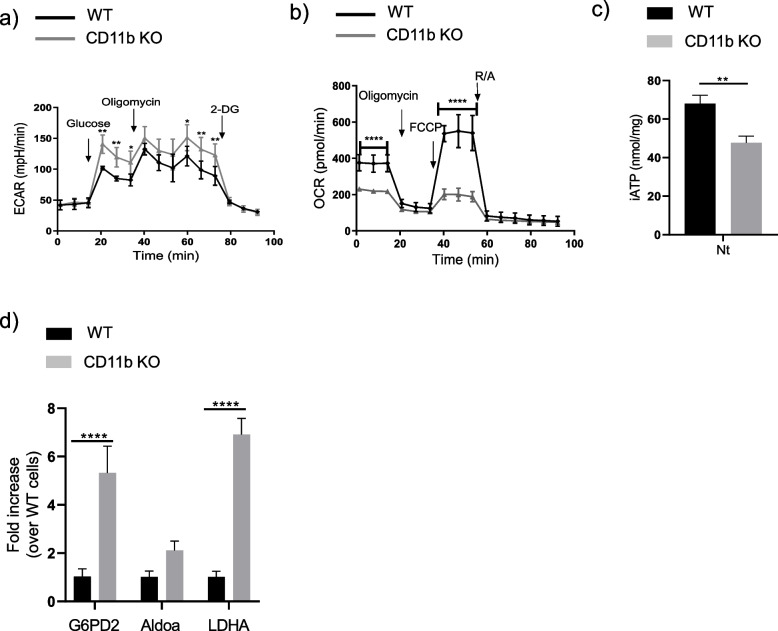
CD11b deficiency favors glycolysis in macrophages. BMDMs from WT or CD11b-deficient mice were isolated and analyzed for (**a**) Time course of real-time changes in the ECAR after Glycolysis Stress Assay. **b** Time-course of real-time changes in the OCR after Cell Mito Stress Assay. **c** iATP levels. **d** Expression of genes involved in glycolysis. G6PD2, Aldoa and LDHA were measured by qRT-PCR and expressed as fold increase over WT cells. Data shown represent mean ± SD with group size *n*=3-4

Using Seahorse technology**,** we analyzed WT and CD11b-deficient macrophages for changes in the rate of extracellular acidification (ECAR), and the mitochondrial rate of oxygen consumption (OCR), as measures of glycolysis and oxidative phosphorylation respectively. CD11b*-*deficient BMDM relied more on glycolysis (higher basal ECAR, Fig. 4a) but less on oxidative phosphorylation (lower basal OCR, Fig. 4b) compared to WT macrophages. Using the same cell conditions, we also measured intracellular ATP (iATP) content and found a significant decrease in iATP in CD11b deficient macrophages compared to WT control cells (Fig. 4c). This latter result is in agreement with the observed shift from oxidative phosphorylation to glycolysis in CD11b-deficient macrophages (as glycolysis produces less ATP than oxidative phosphorylation).

Then, we evaluated whether CD11b deficiency impacted the expression of genes encoding enzymes involved in the glycolysis pathway. Indeed, we observed increased expression of glycolytic enzymes (*i.e.*, glucose 6-phosphate dehydrogenase (G6PD2), aldolase A (Aldoa) and lactate dehydrogenase A (LDHA)) in primed CD11b KO macrophages compared to WT macrophages (Fig. 4d). Altogether, these results show that CD11b modulated the metabolic pathway of macrophages.

**Fig. 5:**
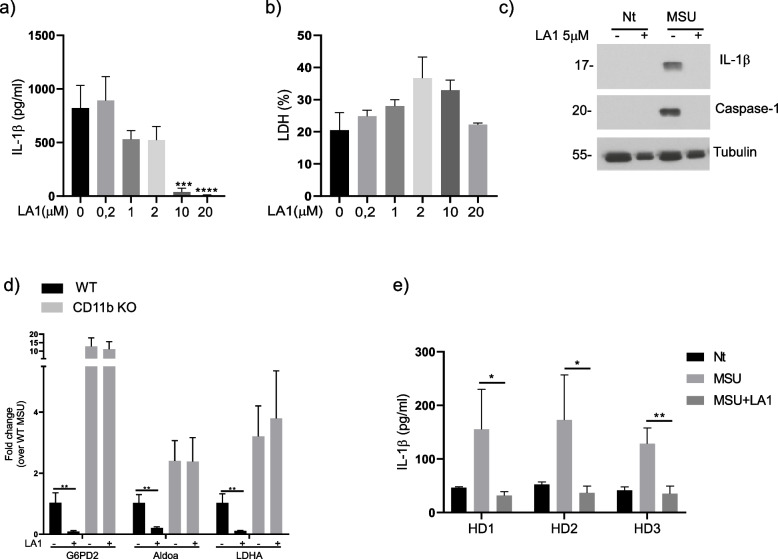
LA1 inhibits NLRP3 inflammasome activation in vitro. BMDMs from WT mice were primed overnight with 100 ng/ml of Pam3Cys. After preincubation with different doses of LA1 for 30 m, cells were stimulated with 500 μg/ml of MSU for 6 h, and supernatants and cell extracts collected (**a**) IL-1β in the supernatants was analyzed by ELISA. **b** Cytotoxicity was evaluated by LDH measurements in the supernatants. **c** IL-1β and caspase-1 in supernatants were analyzed by Western-blot. **d** LA1 modulation of genes involved in glycolysis. Primed WT macrophages were preincubated with 5 µM of LA1 or vehicle and stimulated with 500 μg/ml of MSU for 6h. G6PD2, Aldoa and LDHA were measured by qRT-PCR and expressed as fold change over MSU-treated cells. Black: WT cells, grey: CD11b KO cells. Data shown represent mean ± SD with group size *n*=3. **e** Human IL-1β in peripheral blood mononuclear cells (PBMC) of healthy donors (HD). PBMC were primed overnight with 100 ng/ml of Pam3Cys, preincubated with 5 µM of LA1 or vehicle for 30 min and stimulated with 500 μg/ml of MSU for 6 h. Primed cells, not stimulated with MSU were used as controls (Nt)

We confirmed that in conditions inducing NLRP3 inflammasome activation (*i.e.*, primed macrophages stimulated with MSU crystals), genetic deficiency of CD11b affected glycolytic enzyme expression (G6PD2, Aldoa and LDHA) in a similar way. Indeed, in these conditions, expression of these enzymes was significantly increased in stimulated CD11b KO macrophages compared to WT macrophages (Fig. 5d, compare column 1 to column 3 for each of the enzymes). To gain insights into the involvement of CD11b in cell metabolisms, we performed a metabolomic analysis of primed macrophages stimulated with MSU crystals. CD11b deficiency significantly perturbed some metabolites involved in purine/pyrimidine, glutathione, and amino-acid biosynthesis (Figure S1A). However, the most striking results were observed in lipid metabolism (Figure S1B). CD11b deficiency was associated with a significant increase of several glycerophospholipids (including phosphatidic acid (PA), phosphatidylcholine (PC), lysophosphatidylcholine (LPC), phosphatidylethanolamine (PE), phosphatidylserine (PS) and glycerolipids (Figure S1B).

### The CD11b agonist LA1 inhibits NLRP3 inflammasome activation *in vitro*

It has been reported previously that LA1 modulates the secretion of pro-inflammatory cytokines by monocytes following TLR stimulation [34]. To specifically assess the effect of integrin CD11b activation on NLRP3 inflammasome function, we pretreated primed macrophages with different doses of LA1 followed by MSU stimulation and measured IL-1β secretion. In these conditions, LA1 treatment inhibited MSU-induced IL-1β secretion in a concentration-dependent manner (Fig. 5a). Importantly, in the same experimental conditions LA1 had no effect on cytotoxicity, as measured by lactate dehydrogenase (LDH) release (Fig. 5b).

To further confirm that decreased IL-1β secretion was caused by inhibitory actions of LA1 on caspase-1 activation and IL-1β processing, we analyzed caspase-1/IL-1β protein expression by Western-blot. In line with the IL-1β ELISA results, LA1 inhibited both caspase-1 activation and IL-1β processing that were induced by MSU crystals, as evidenced by reduced active p20 caspase-1 and active p17 IL-1β levels in cell supernatants, respectively (Fig. 5c). Additionally, we also found that LA1 could reduce the expression of the previously identified glycolytic enzymes (G6PD2, Aldoa, LDHA) in WT macrophages but as expected for a CD11b agonist, did not impact their expression in CD11b-deficient macrophages (Fig. 5d, compare column 3 to column 4 for each of the enzymes)). Finally, we investigated the effect of LA1 using human peripheral blood mononuclear cells (PBMC) isolated from three independent healthy donors. Consistent with our previous results on murine macrophages, LA1 inhibited IL-1β secretion in the supernatant of MSU- stimulated healthy human PBMC (Fig. 5e).

### Administration of LA1 reduces the severity of MSU-induced gouty arthritis in mice.

**Fig. 6:**
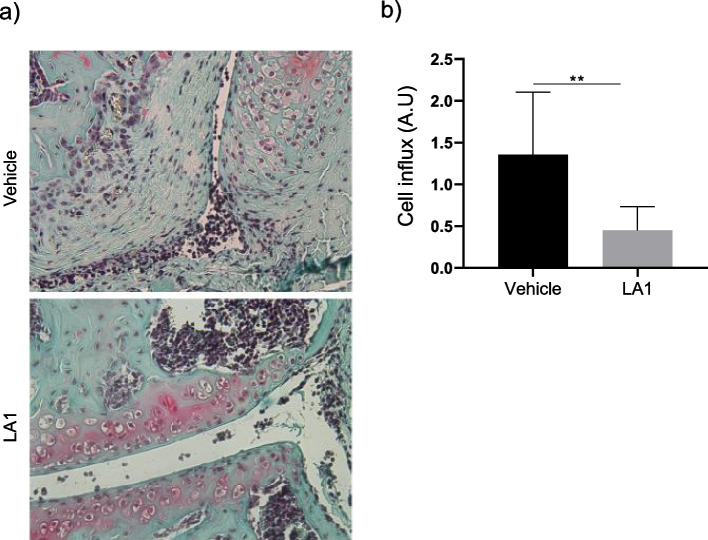
LA1 inhibits MSU-induced joint inflammation in a murine gouty arthritis. **a** Representative images of knees from H&E-stained histological knee sections from vehicle-treated and LA1-treated WT mice. **b** Mean ± SD of cell influx after 6 h of treatment (*n*=7) per group

To explore the anti-inflammatory effect of LA1 *in vivo*, we investigated whether administration of LA1 (injected *i.v.* 30 min before intra-articular administration of crystals) suppresses joint inflammation in WT mice. Histological analyses of the knee joints showed decreased influx of inflammatory cells in the synovial cavity (Figure 6a). This reduction was significant as shown by the scoring of cell influx in knee joints from LA1-treated versus vehicle-treated mice (Fig. 6b).

## Discussion

This study provides the first evidence that CD11b deficiency aggravates the severity of MSU-induced gouty arthritis through increasing IL-1β maturation and enhancing infiltration of proinflammatory cells in the knee joint. The *in vivo* data are supported by the increased production of IL-1β and IL-6 in CD11b-deficient macrophages and THP-1 cells *in vitro* following MSU stimulation. This finding is consistent with our previously reported anti-inflammatory function of CD11b integrin in different cell types [23, 29, 43].

The mechanisms involved in exacerbated NLRP3 inflammasome activation upon MSU crystal stimulation have been explored. Phagocytosis is the initial step in crystal-induced inflammasome activation. We found decreased phagocytic activity toward MSU in CD11b-deficient macrophages, suggesting that MSU crystals bind directly or in directly to CD11b. Indeed, a previous study has reported that CD11b could mediate this effect indirectly by enhancing the engagement of CD16 with MSU crystals [44]. However, as phagocytosis is reduced in CD11b deficient cells, it cannot explain the increased inflammasome activation in these cells. We also ruled out the involvement of ROS in the exacerbated NLRP3 inflammasome activation, as an explanatory mechanism, as ROS levels were similar in MSU-stimulated WT and CD11b^-/-^ cells.

A metabolic shift towards aerobic glycolysis is a hallmark of NLRP3 inflammasome activation. For instance, LPS was shown to induce metabolic reprogramming of macrophages into a highly glycolytic state resulting in increased succinate production that increases the expression of IL-1β [45]. Our data demonstrated that the absence of CD11b in primed macrophages leads to reduced oxygen consumption, increased glycolysis, consistent with previous findings that showed a critical role of glycolysis in NLRP3 inflammasome activation after macrophage priming and MSU stimulation [46]. The question remained as to what is the signaling cascade(s) immediately downstream of CD11b that leads to this metabolic shift.

In the present study, metabolomics analysis provided information about CD11b-dependent metabolic pathways potentially involved in NLRP3 inflammasome activation. The majority of the metabolites significantly down- or up-regulated in MSU-stimulated WT versus CD11b-deficient macrophages are lipids. In particular, upon MSU-induced NLRP3 inflammasome activation, several glycerophospholipids (PA, PC, PE, PS and LPC) were increased in CD11b-deficient macrophages. Interestingly, LPC has a wide range of proinflammatory activities and has been previously reported to induce the secretion of IL-1β, via NLRP3 inflammasome activation [47]. Saturated fatty acids such as palmitic acid and stearic acid (C18:0) can also activate the NLRP3 inflammasome [48]. Interestingly, a strong association between gout, NlLRP3 inflammasome activation and metabolic syndrome is becoming increasingly evident [49]. Whether dysregulated NLRP3 inflammasome activation in CD11b-deficient macrophages is accounted for by metabolic changes (metabolic shift towards glycolysis, lipid metabolism and overproduction of specific metabolites) remains to be established.

Our finding identified CD11b integrin as a negative regulator to prevent uncontrolled activation of NLRP3 inflammasome and the overproduction of this proinflammatory cytokine in macrophages stimulated with MSU. The regulatory role of CD11b in inflammasome activation has been evidenced in several studies, with contrasting results. Ligation of the integrin by pre-opsonized virulent *F. tularensis* limits inflammasome activation via reduced pro IL-1β expression in human phagocytic cells [50]. In a murine model of sepsis induced by methicillin-resistant Staphylococcus aureus infection, CD11b deficiency resulted in increased susceptibility to sepsis by enhancing the pro-inflammatory activities of macrophages. Indeed, upon bacterial infection, both bone marrow-derived macrophages and peritoneal macrophages lacking CD11b expressed significantly higher levels of proinflammatory cytokines mRNA, including that for IL-1β [51]. In accordance with a negative regulatory role of CD11b in IL-1β production, in an optic nerve crush model, CD11b deficient retinae exhibited exacerbated microglial activation, as well as more proinflammatory cytokines [52].However, in other studies CD11b seems to favor NLRP3 inflammasome activation. Indeed, CD11b contributed to β-glucan–induced inflammasome response, and accordingly upon β-glucan stimulation no IL-1β was induced in CD11b-deficient cells [53]. Similarly, CD11b deficiency reduced NLRP3 inflammasome activation in microglial cells in a model of Parkinson’s disease [54]. Altogether, the role of CD11b in NLRP3 inflammasome activation seems to be trigger- and/or cell-specific.

Several NLRP3 inflammasome inhibitors have been identified [10] acting via different mechanisms. We report here that LA1 behaves as a NLRP3 inflammasome inhibitor, reducing IL-1β secretion both in primary murine and human cells stimulated with MSU crystals and in a murine gout model *in vivo*. In the past, LA1 has been described to promote CD11b activation to limit overactivation of macrophages [34], essentially by inhibiting proIL-1β expression.

## Conclusion

In summary, our work shows that upon MSU stimulation, CD11b limits inflammation in pro-inflammatory macrophages and leads to metabolic changes. As a negative regulator of inflammasome signaling, CD11b can be an attractive therapeutic target for the treatment of excessive inflammation in gout.

### Supplementary Information

Supplementary Material 1.

Supplementary Material 2.

Supplementary Material 3: Metabolomic analysis of MSU stimulated WT and CD11b deficient macrophages. A),B) The effect of CD 11 b deficiency on metabolites was studied in primed untreated cells (NT) or upon MSU crystal stimulation (MSU). The heat map represents the mean ratio of metabolites in WT versus CD11b KO cells Red colors indicate a decrease metabolite level in WT whereas green color an increased one (as indicated by negative or positive Log 2 FC) Significant changes are indicated with *.

## Data Availability

No datasets were generated or analysed during the current study.

## Abbreviations

MSU: Monosodium urate
NLRP3: Nucleotide-binding oligomerization domain-like receptor protein 3
TLR: Toll-like receptors
ROS: Reactive oxygen species
HMGB-1: High mobility group box protein 1
LA1: Leukadherin‐1
BMDMs: Bone marrow derived macrophages
PBMCs: Peripheral blood mononuclear cells
qRT-PCR: Quantitative Real Time PCR
ECAR: Real-time extracellular acidification rate
OCR: Oxygen consumption rate
FCCP: Carbonyl cyanide-4-(trifluoromethoxy) phenylhydrazone
Rot/AA: Rotenone/antimycin A
LDH: Lactate dehydrogenase
G6PD2: Glucose 6-phosphate dehydrogenase
Aldoa: Aldolase A
LDHA: Lactate dehydrogenase A
PA: Phosphatidic acid
PC: Phosphatidylcholine
LPC: Lysophosphatidylcholine
PE: Phosphatidylethanolamine
PS: Phosphatidylserine
