# Supplementary figures and images for: The integrin CD11b inhibits MSU-induced NLRP3 inflammasome activation in macrophages and protects mice against MSU-induced joint inflammation
Source: Arthritis Res Ther. 2024 Jun 11;26:119. doi: 10.1186/s13075-024-03350-5 (PMC11165854; doi:10.1186/s13075-024-03350-5)

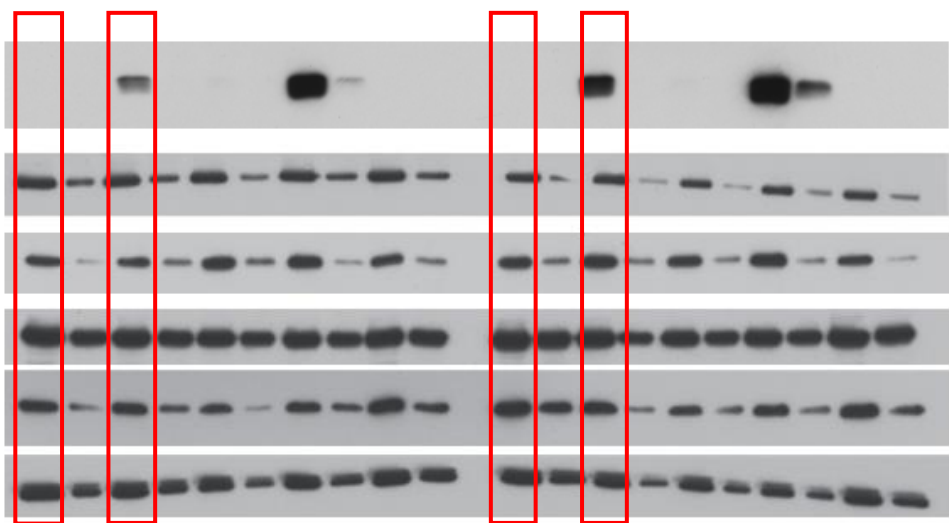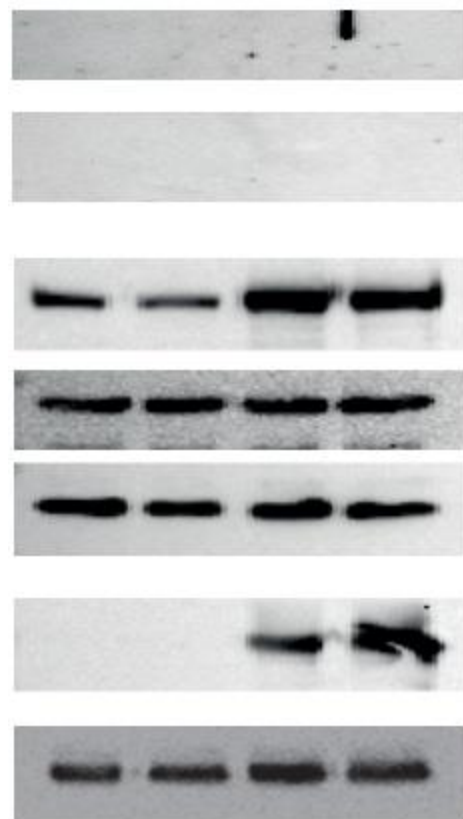

Supplement: Supplementary file 1 — Supplementary Material 1. [file 13075_2024_3350_MOESM1_ESM.pdf]

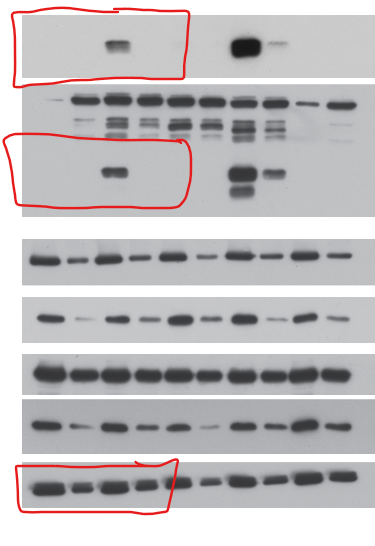

Supplement: Supplementary file 2 — Supplementary Material 2. [file 13075_2024_3350_MOESM2_ESM.png]
