# Supplementary material for: The integrin CD11b inhibits MSU-induced NLRP3 inflammasome activation in macrophages and protects mice against MSU-induced joint inflammation
Source: Arthritis Res Ther. 2024 Jun 11;26:119. doi: 10.1186/s13075-024-03350-5 (PMC11165854; doi:10.1186/s13075-024-03350-5)
Supplement: Supplementary file 3 — Supplementary Material 3: Metabolomic analysis of MSU stimulated WT and CD11b deficient macrophages. A),B) The effect of CD 11 b deficiency on metabolites was studied in primed untreated cells (NT) or upon MSU crystal stimulation (MSU). The heat map represents the mean ratio of metabolites in WT versus CD11b KO cells Red colors indicate a decrease metabolite level in WT whereas green color an increased one (as indicated by negative or positive Log 2 FC) Significant changes are indicated with *. [file 13075_2024_3350_MOESM3_ESM.pptx]

## Slide 1
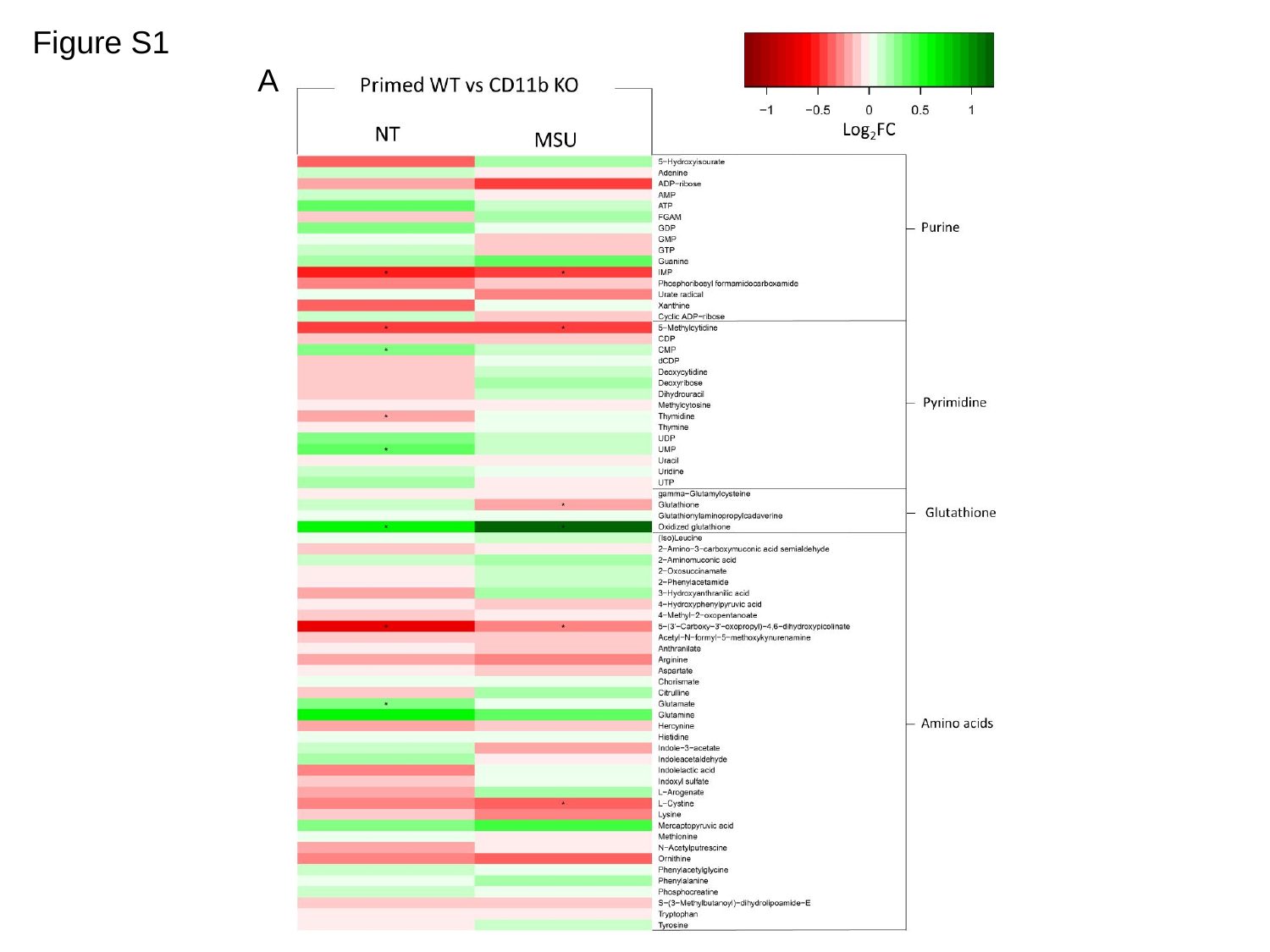

Figure S1
A

## Slide 2
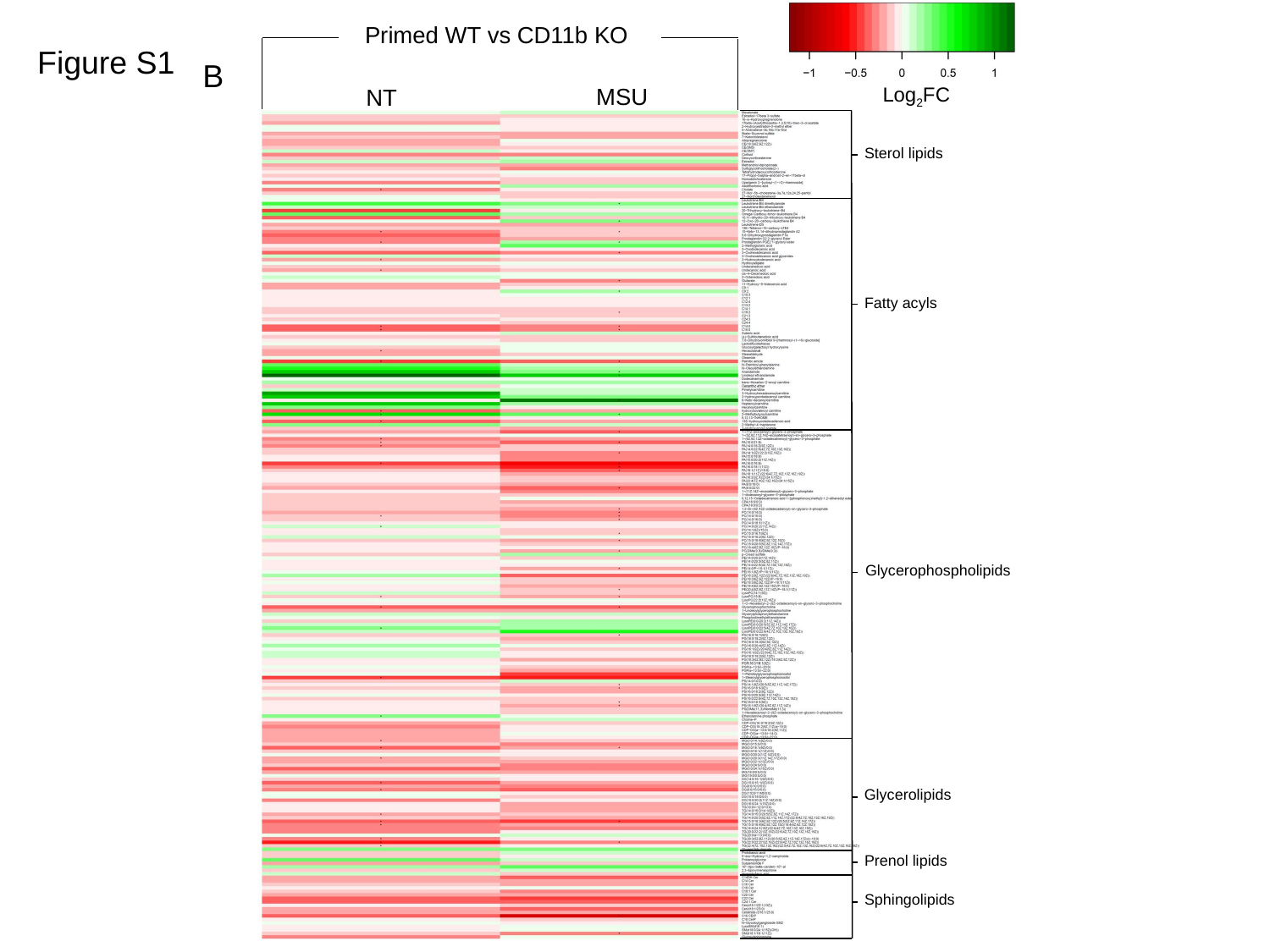

B
Primed WT vs CD11b KO
MSU
 Log2FC
NT
Sterol lipids
Fatty acyls
Glycerophospholipids
Glycerolipids
Prenol lipids
Sphingolipids
Figure S1

## Slide 3
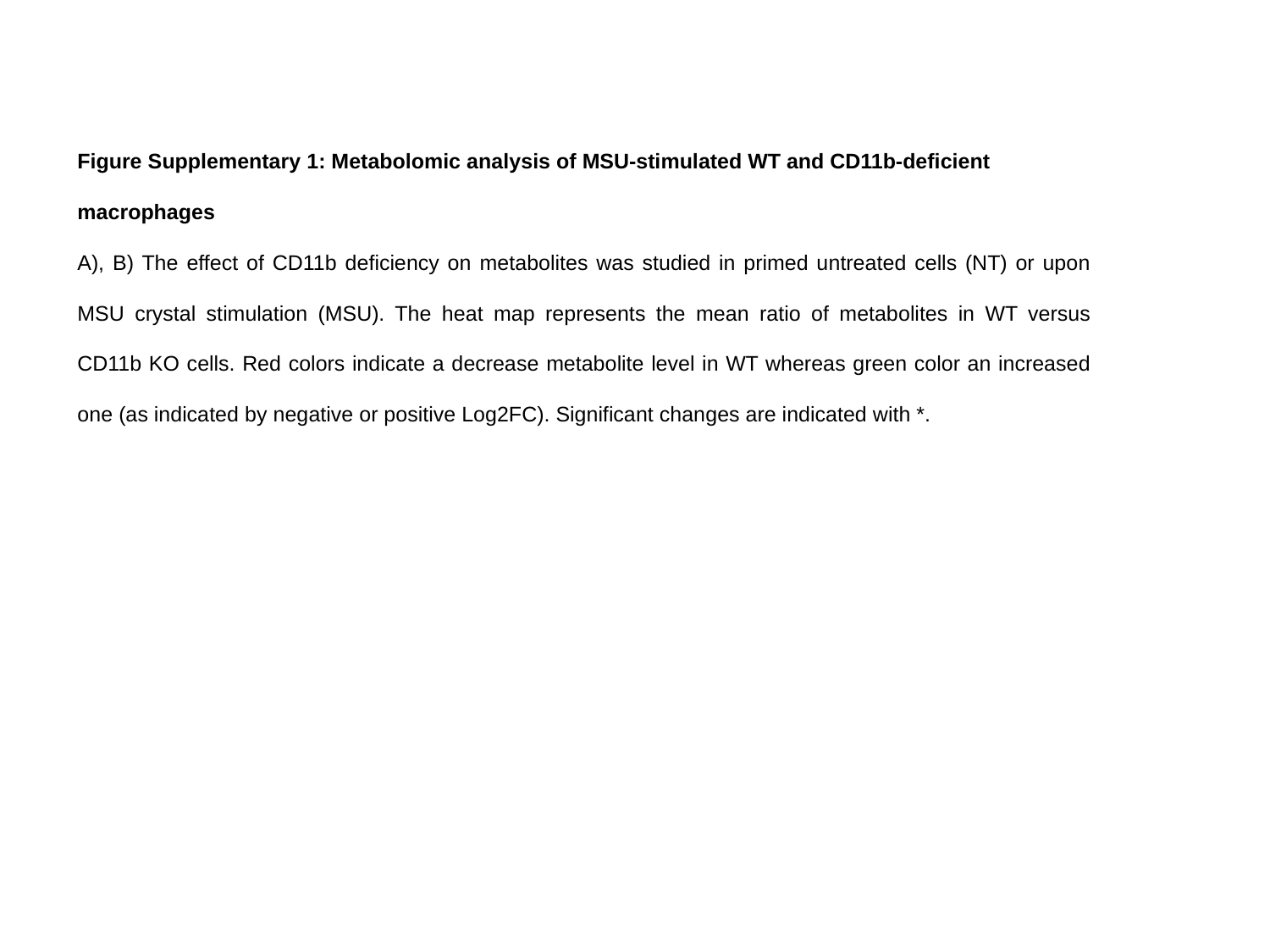

Figure Supplementary 1: Metabolomic analysis of MSU-stimulated WT and CD11b-deficient macrophages
A), B) The effect of CD11b deficiency on metabolites was studied in primed untreated cells (NT) or upon MSU crystal stimulation (MSU). The heat map represents the mean ratio of metabolites in WT versus CD11b KO cells. Red colors indicate a decrease metabolite level in WT whereas green color an increased one (as indicated by negative or positive Log2FC). Significant changes are indicated with *.
